# Supplementary material for: Genomic epidemiological models describe pathogen evolution across fitness valleys
Source: Sci Adv. 2022 Jul 13;8(28):eabo0173. doi: 10.1126/sciadv.abo0173 (PMC9278859; doi:10.1126/sciadv.abo0173)
Supplement: Supplementary file 1 — Figs. S1 to S8 Tables S1 to S3 [file sciadv.abo0173_sm.pdf]

Science Advances
AAAS

Supplementary Materials for

Genomic epidemiological models describe pathogen evolution across fitness valleys

Pablo Cárdenas *et al.*

Corresponding author: Pablo Cárdenas, pcarden@mit.edu

Sci. Adv. **8**, eabo0173 (2022)
DOI: 10.1126/sciadv.abo0173

This PDF file includes:

Figs. S1 to S8
Tables S1 to S3

Figures S1 – S8

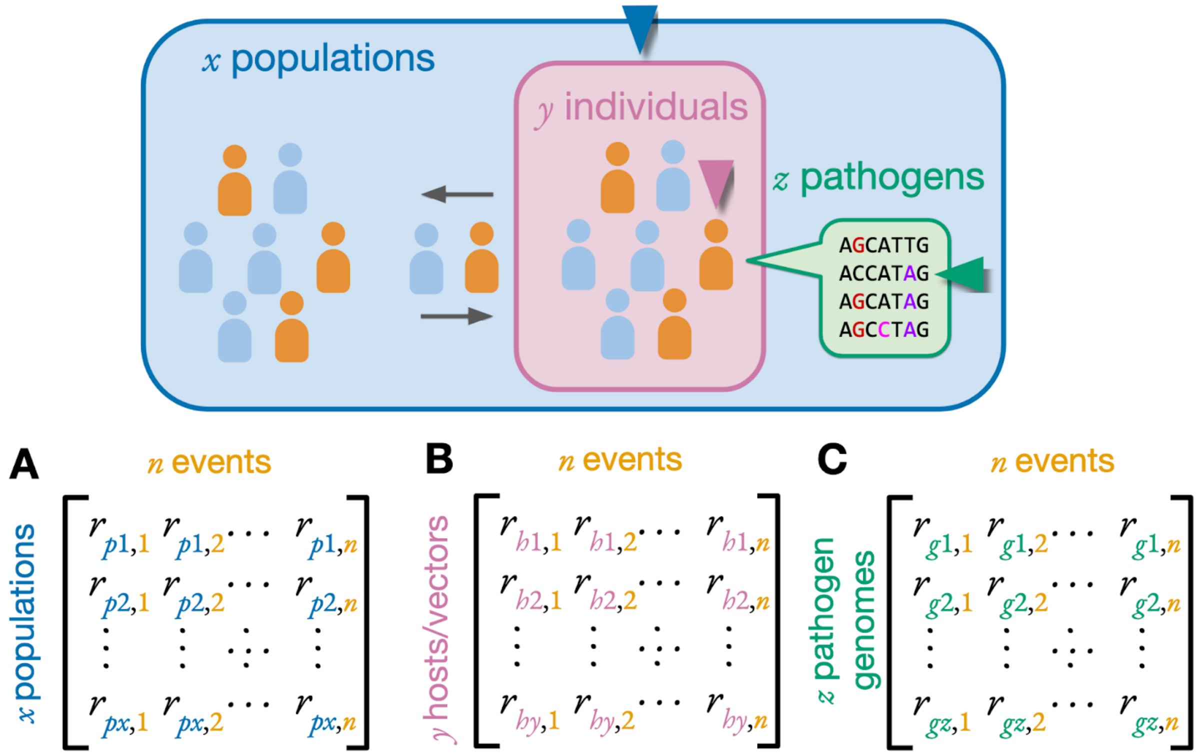

Supplementary Fig. S1. Opqua uses different levels of rate matrices to sample events. (A) A matrix contains the rates r_p of n different events for x different populations within the model. A given event type and population are sampled randomly based on the rate of each event-population combination. **(B)** Each population contains matrices with the event rates r_i for all y individual hosts and vectors it contains. A host/vector is sampled randomly based on the rates of the chosen event type for all host/vectors in the chosen population. **(C)** Finally, a similar process occurs within hosts and vectors to randomly sample pathogens within them, based on how their z different genomes affect the rates r_g of the chosen event. Some events involve sampling an additional population (migration or inter-population contact), host/vector (inter- or intra-population contact), or pathogen (recombination). Once event type, population(s), host(s) and/or vector(s), and pathogen(s) have been chosen in this manner, the state of the model is adjusted according to the event, and the relevant rate changes are propagated upward from within the pathogens affected to the host(s), vector(s), populations, and overall model they are in.

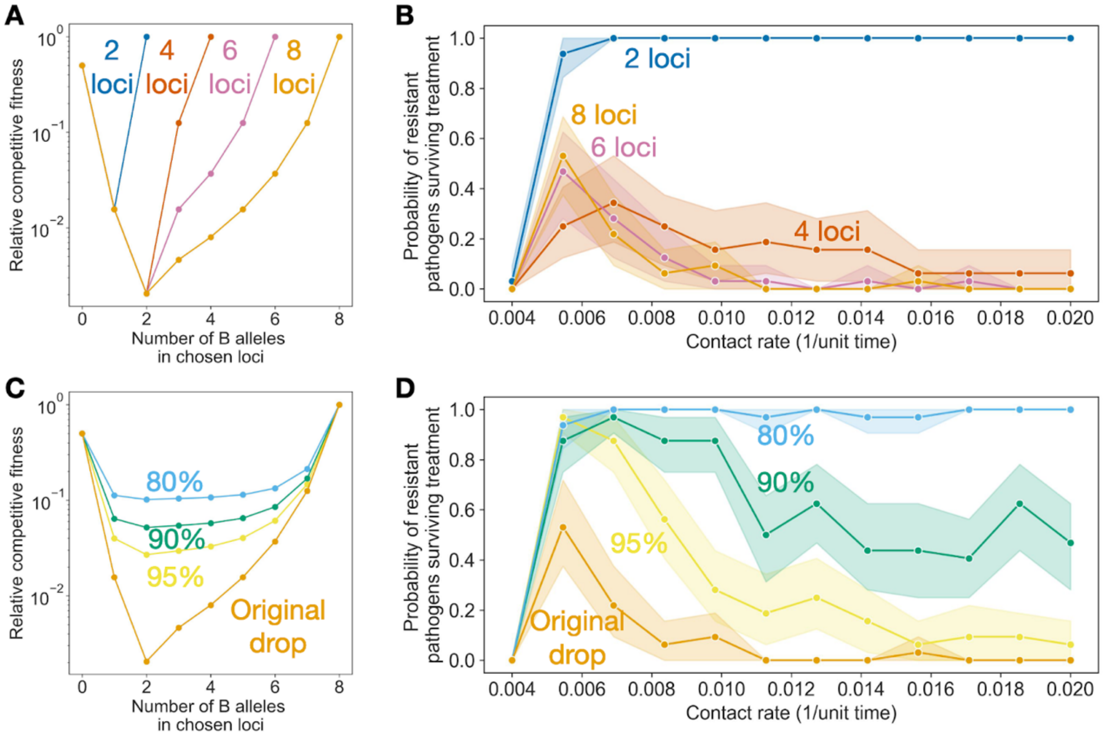

Supplementary Fig. S2. Length and steepness of the fitness valley affect the impact of competition on evolution. We constructed a model to simulate stochastic tunneling of a pathogen across a fitness valley based on genomes with eight bi-allelic loci, as portrayed in Fig. 2. **(A)** By focusing on a subset of loci in each genome, we can vary the length of the fitness valley. **(B)** Longer fitness valleys favor the evolution and survival of pathogens in low transmission environments, as more loci provide more alternative evolutionary paths through the valley but are inhibited by high intra-host competition. In short fitness valleys, the number of paths becomes more restricted while the effect of competition is lessened, favoring environments with increased transmission. **(C)** By adjusting the fitness cost of intermediate mutants, we can vary the steepness and depth of the fitness valley. **(D)** Steep, deep valleys increase the effect of intra-host competition, favoring the evolution and survival of pathogens in low transmission environments more heavily.

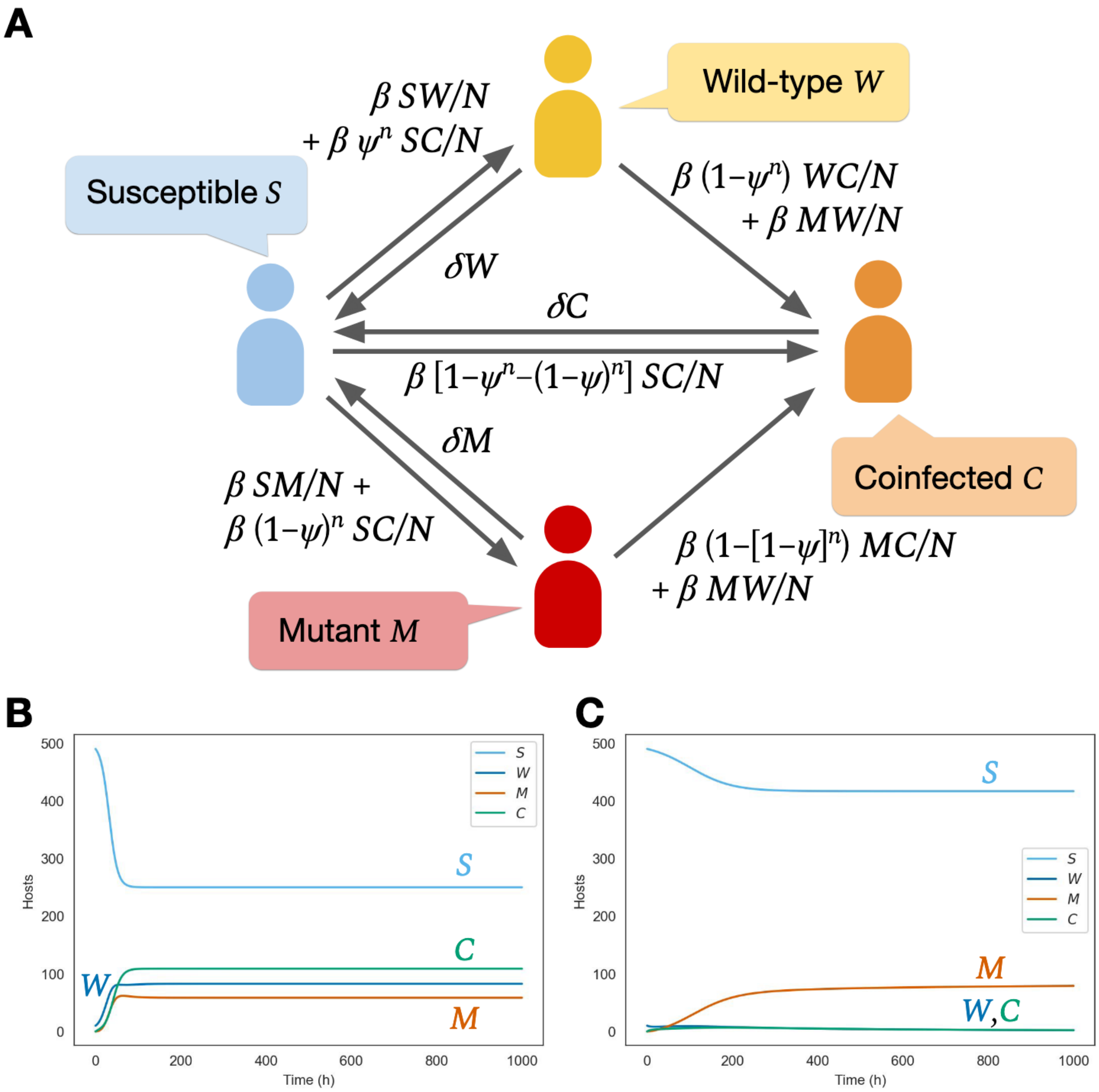

Supplementary Fig. S3. Compartment model structure allows deterministic simulation of dynamics of two strains of pathogens. (A) The model consists of compartments for hosts infected with no pathogens (*S*), wild-type pathogens only (*W*), mutant pathogens only (*M*), or coinfected with both kinds of pathogens (*C*). Transitions between compartments are determined by host recovery rate δ, contact rate β, mutation rates from wild-type to mutants μ_1 and vice-versa μ_2, inoculum size *n*, and the probability of wild-type pathogens with higher fitness outcompeting mutants in intra-host competition ψ. **(B)** At high transmission intensities (β=0.2), unfit mutants (ψ=0.67) constitute a small portion of the total population, as shown throughout the rest of this work. **(C)** At low transmission intensities (β=0.12), unfit mutants show greater prevalence in the population.

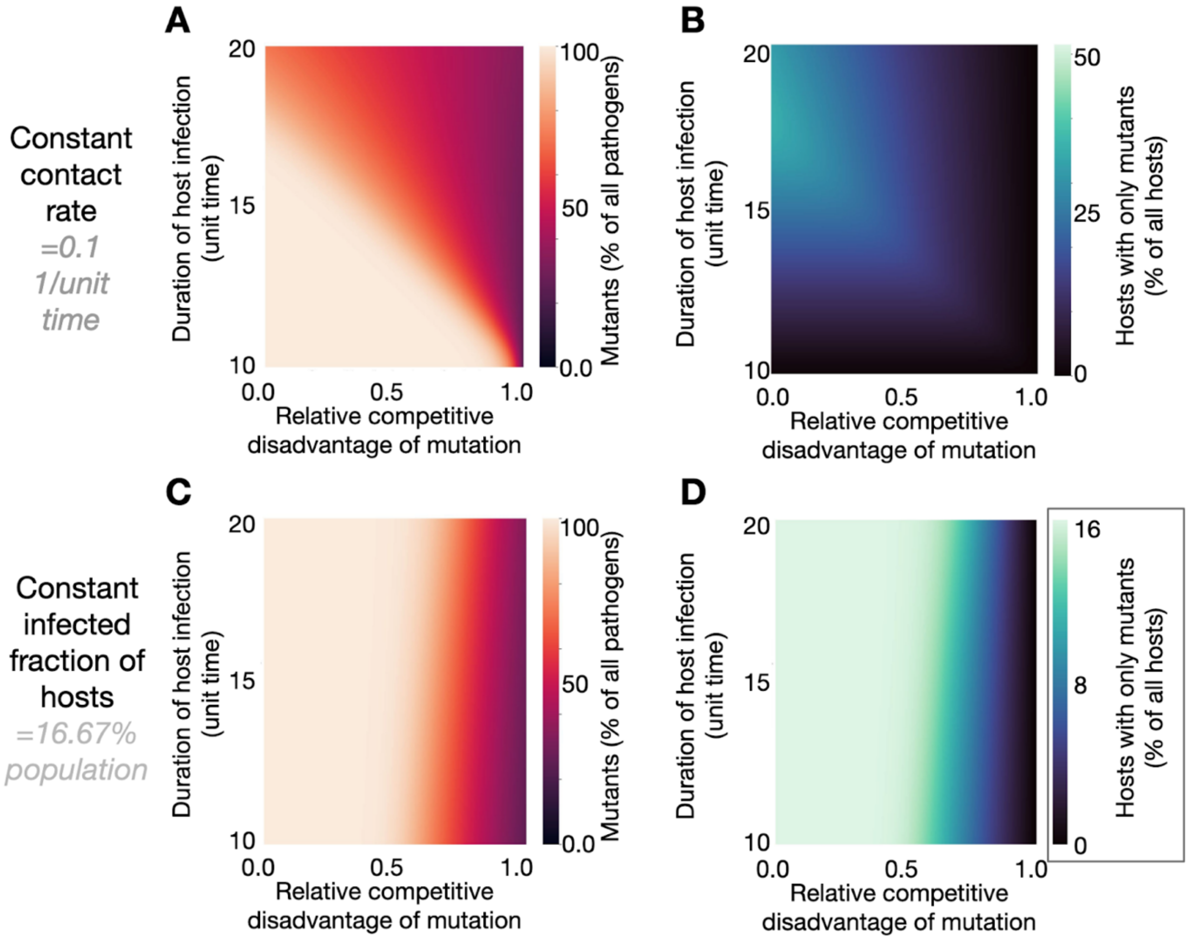

Supplementary Fig. S4. Duration of infection affects mutant fraction primarily through competition for free hosts, as with contact rate. Using the ordinary differential equation model described, we vary the duration of infection (equivalent to 1/recovery rate) while keeping the contact rate constant such that the range of steady-state infected hosts sampled is equivalent to those in Figure 3B and 3C. The resulting **(A)** mutant fractions of pathogens and **(B)** mutant-only fractions of infections are similar to those shown when varying contact rates in Figure 3B and 3C. Small differences in the scale of the effects are due to the fact that at high durations of infection, mutants are removed less frequently through recovery. This can be seen when varying the duration of infection while keeping the fraction of infected hosts constant by simultaneously varying the contact rate. While biologically unlikely to happen, this shows a small increase in fraction of **(C)** mutants among pathogens and **(D)** mutant-only infections among hosts (note change in color scale).

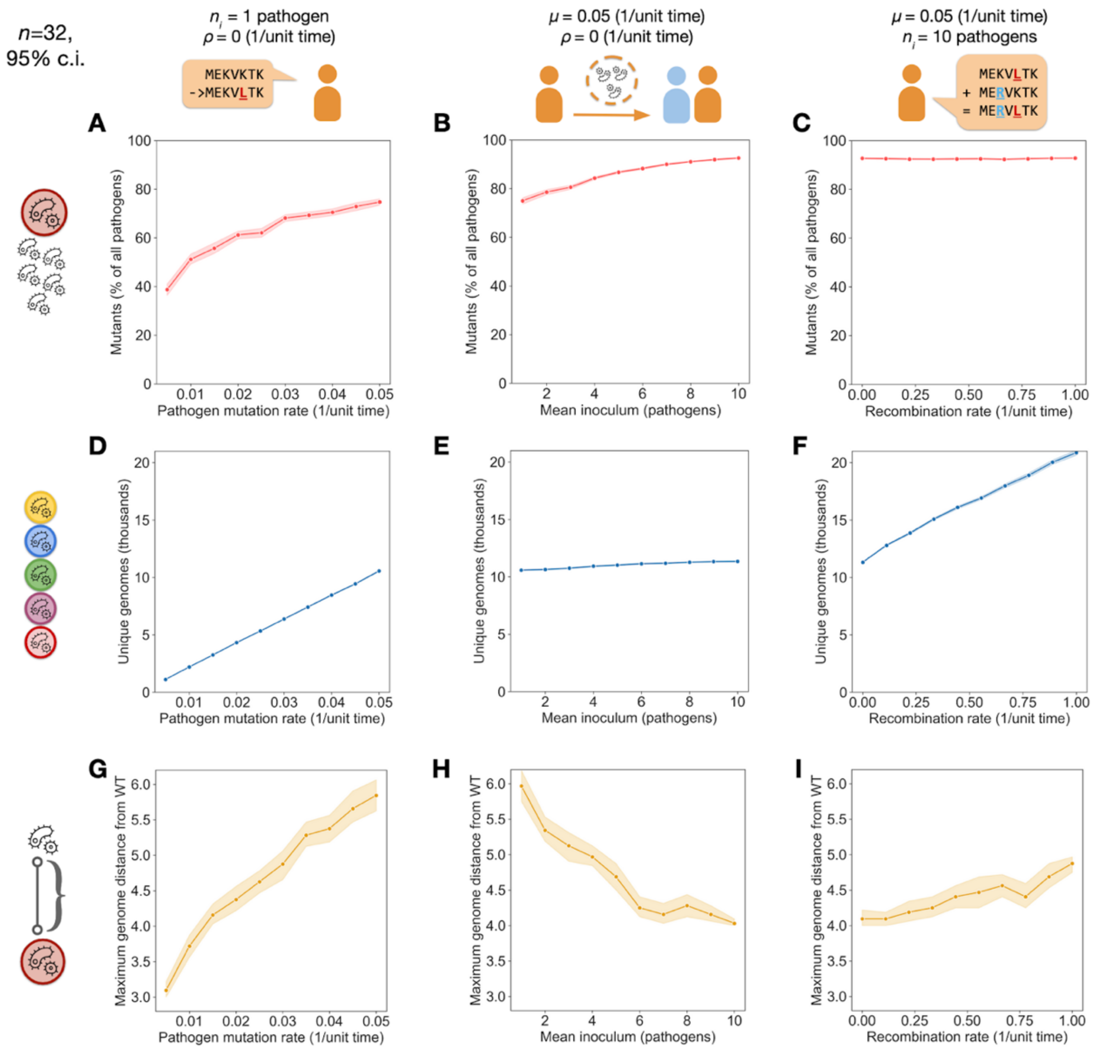

Supplementary Fig. S5. Pathogen biology affects the distribution and dimensions of their evolution for host-host direct transmission. The effects of mutation rate, inoculum size from hosts, and recombination rates are analogous to those observed within hosts for a vector-borne model (Fig. 5). The fraction of mutants in the pathogen population is increased by both **(A)** high mutation rates (μ) and **(B)** high mean inoculum sizes (n_i), but is unaffected by **(C)** recombination rates (ρ). **(D)** The number of unique pathogen genomes in the simulation, which we treat as a measure of the "width" of evolutionary space explored, increases with high mutation rates, but less so with **(E)** high mean inoculum size. **(F)** Greater recombination increases the number of unique pathogen genomes. Lastly, **(G)** increased mutation rate increases the "depth" of evolutionary space explored by pathogens, measured as the maximum Hamming distance of mutant genomes from the initial wild-type sequence. **(H)** Low inoculum size increases the probability of transmitting mutants without wild-type (WT) competitors, allowing for greater depth in the evolutionary space explored. **(I)** High recombination rates increase depth of evolutionary space, notably without affecting the fraction of mutants in the pathogen population.

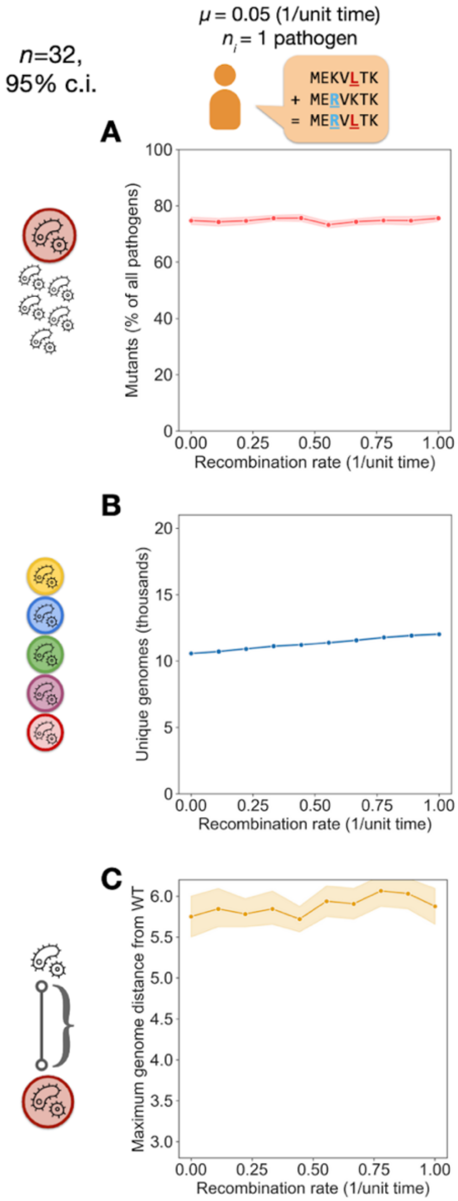

Supplementary Fig. S6. Recombination depends on inoculum size to increase evolutionary distance. By keeping the mutation rate (μ) constant and reducing the mean inoculum size (n_i) to 1 in a host-host transmission model with a descending fitness landscape, we can see the effects of recombination on pathogen genome evolution with low inoculums. **(A)** As before, increasing recombination rate does not increase the mutant fraction of pathogens. However, the lower inoculum greatly reduces the effect of recombination on **(B)** the number of unique pathogen genomes explored and **(C)** the maximum Hamming distance from the wild-type pathogen genotype. Neither of these shows increases on the same scale seen for the higher mean inoculum size n_i=10 in Fig. S3.

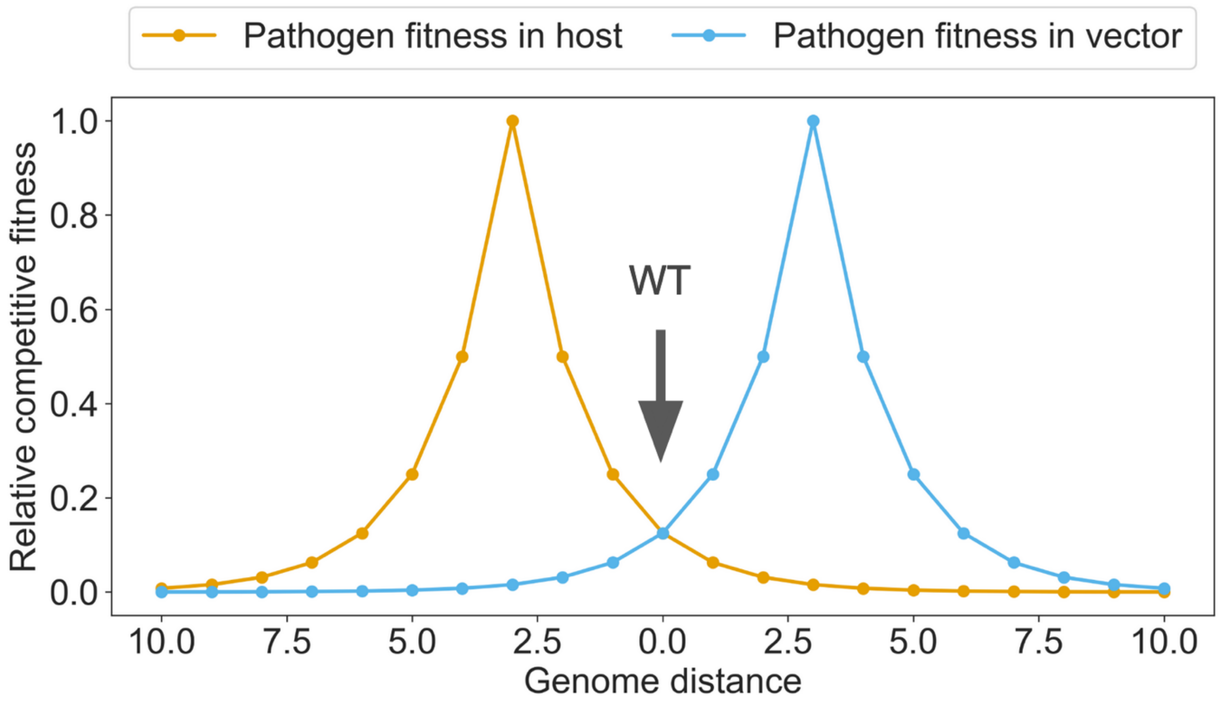

Supplementary Fig. S7. Separate fitness landscapes within hosts and vectors can be used to study pathogen evolution in Opqua. Two fitness functions are devised with exponentially decaying fitness around distinct, optimal genome sequences. These optimal genomes constitute separate fitness peaks for pathogens within hosts and vectors. Each fitness peak is at Levenshtein distance of six mutations from the other. The wild-type (WT) genome sequence used to initiate simulations lies halfway between both peaks, three mutations from each.

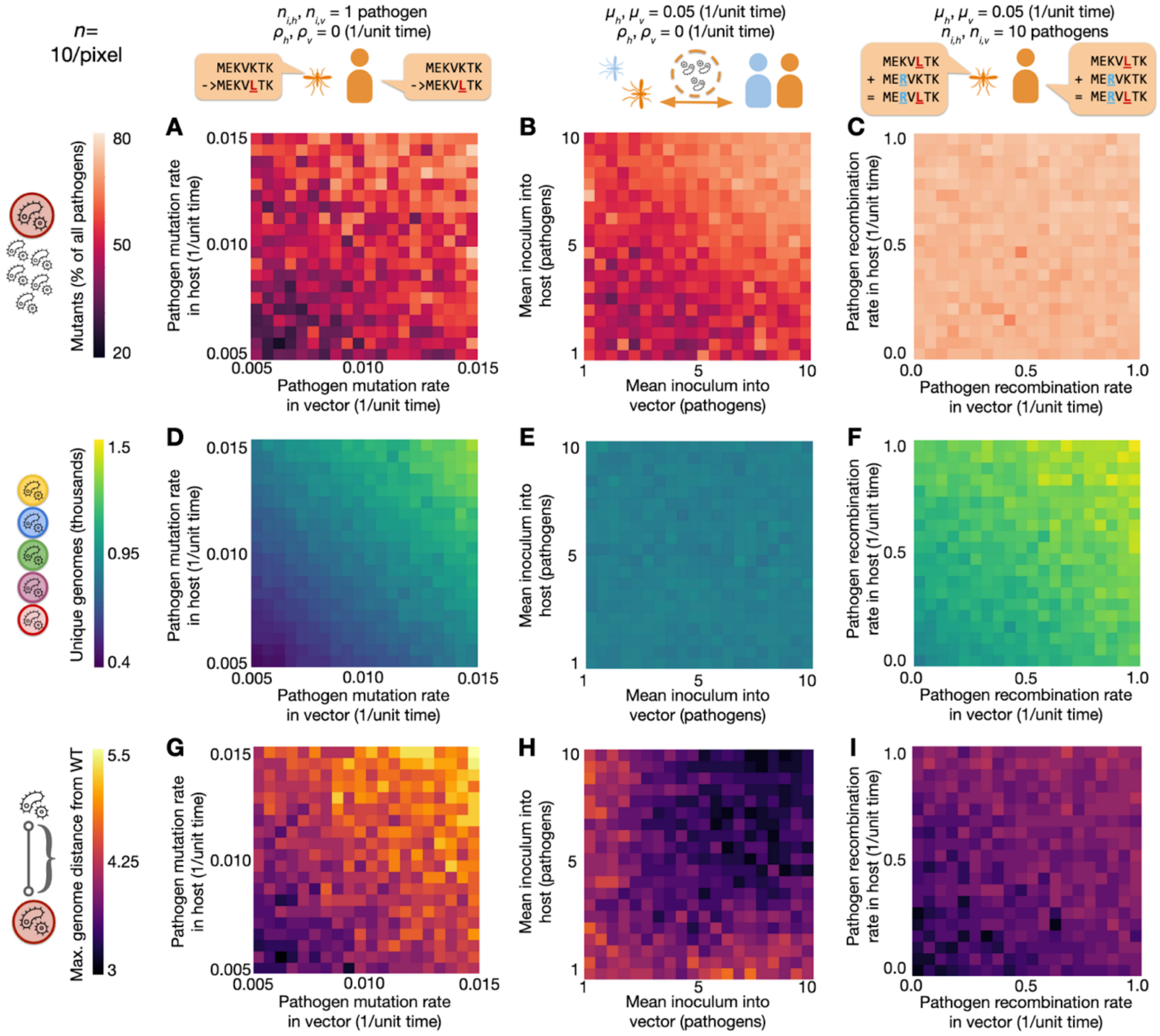

Supplementary Fig. S8. Conflicting fitness landscapes in different life stages generate symmetric distributions of evolution within hosts and vectors. We varied mutation rates, inoculum size, and recombination rates in hosts and vectors as done in the simulations shown on Fig. 5, with the addition of selection within both hosts and vectors (as shown in Fig. S7). The resulting heatmaps show distributions of **(A–C)** mutant pathogens, **(D–F)** unique genomes explored, and **(G–I)** maximum genome distance from the wild-type genome that are symmetric across the diagonal, and correspond to the average of each respective graph on Fig. 5 and its diagonal reflection. This is expected, given the same population bottlenecks described in the main text for the model with only selection in hosts are now present with symmetric selection pressures across the life cycle.

Table S1: Default Opqua parameters for host-host transmission models*

**Parameter name**	**Default value**
num_loci	10
possible_alleles	'ATCG'
fitnessHost	(lambda g: 1)
contactHost	(lambda g: 1)
receiveContactHost	(lambda g: 1)
mortalityHost	(lambda g: 1)
natalityHost	(lambda g: 1)
recoveryHost	(lambda g: 1)
migrationHost	(lambda g: 1)
populationContactHost	(lambda g: 1)
receivePopulationContactHost	(lambda g: 1)
mutationHost	(lambda g: 1)
recombinationHost	(lambda g: 1)
fitnessVector	(lambda g: 1)
contactVector	(lambda g: 1)
receiveContactVector	(lambda g: 1)
mortalityVector	(lambda g: 1)
natalityVector	(lambda g: 1)
recoveryVector	(lambda g: 1)
migrationVector	(lambda g: 1)
populationContactVector	(lambda g: 1)
receivePopulationContactVector	(lambda g: 1)

mutationVector	(lambda g: 1)
recombinationVector	(lambda g: 1)
contact_rate_host_vector	0
transmission_efficiency_host_vector	0
transmission_efficiency_vector_host	0
contact_rate_host_host	2.00E-01
transmission_efficiency_host_host	1
mean_inoculum_host	1.00E+01
mean_inoculum_vector	0
recovery_rate_host	1.00E-01
recovery_rate_vector	0
mortality_rate_host	0
mortality_rate_vector	0
recombine_in_host	1.00E-04
recombine_in_vector	0
num_crossover_host	1
num_crossover_vector	0
mutate_in_host	1.00E-06
mutate_in_vector	0
death_rate_host	0
death_rate_vector	0
birth_rate_host	0
birth_rate_vector	0
vertical_transmission_host	0

vertical_transmission_vector	0
inherit_protection_host	0
inherit_protection_vector	0
protection_upon_recovery_host	None
protection_upon_recovery_vector	None

*For a detailed description of each parameter, consult the documentation at https://github.com/pablocarderam/opqua#newsetup

Table S2: Default Opqua parameters for vector-borne transmission models*

**Parameter name**	**Default value**
num_loci	10
possible_alleles	'ATCG'
fitnessHost	(lambda g: 1)
contactHost	(lambda g: 1)
receiveContactHost	(lambda g: 1)
mortalityHost	(lambda g: 1)
natalityHost	(lambda g: 1)
recoveryHost	(lambda g: 1)
migrationHost	(lambda g: 1)
populationContactHost	(lambda g: 1)
receivePopulationContactHost	(lambda g: 1)
mutationHost	(lambda g: 1)
recombinationHost	(lambda g: 1)
fitnessVector	(lambda g: 1)
contactVector	(lambda g: 1)

receiveContactVector	(lambda g: 1)
mortalityVector	(lambda g: 1)
natalityVector	(lambda g: 1)
recoveryVector	(lambda g: 1)
migrationVector	(lambda g: 1)
populationContactVector	(lambda g: 1)
receivePopulationContactVector	(lambda g: 1)
mutationVector	(lambda g: 1)
recombinationVector	(lambda g: 1)
contact_rate_host_vector	2.00E-01
transmission_efficiency_host_vector	1
transmission_efficiency_vector_host	1
contact_rate_host_host	0
transmission_efficiency_host_host	0
mean_inoculum_host	1.00E+02
mean_inoculum_vector	1.00E+00
recovery_rate_host	1.00E-01
recovery_rate_vector	1.00E-01
mortality_rate_host	0
mortality_rate_vector	0
recombine_in_host	0
recombine_in_vector	1.00E-04
num_crossover_host	0
num_crossover_vector	1

mutate_in_host	1.00E-06
mutate_in_vector	0
death_rate_host	0
death_rate_vector	0
birth_rate_host	0
birth_rate_vector	0
vertical_transmission_host	0
vertical_transmission_vector	0
inherit_protection_host	0
inherit_protection_vector	0
protection_upon_recovery_host	None
protection_upon_recovery_vector	None

*For a detailed description of each parameter, consult the documentation at https://github.com/pablocarderam/opqua#newsetup

Table S3: Parameters used for two-strain compartment model

Parameter name	Description	Value
δ	host recovery rate	1.00E-01*
β	host contact rate	1.10E-01*
φ	probability of wild-type pathogens outcompeting mutants in intra-host competition	1/(2-0.1)*
n_i	size of inoculum	1
μ_1	mutation rate from wild-type to mutant	5.00E-02
μ_2	mutation rate from mutant to wild-type	0

N	total population size	5.00E+02

*Parameters vary according to simulation
